# The Safety, Efficacy, and Tolerability of Microbial Ecosystem Therapeutic-2 in People With Major Depression and/or Generalized Anxiety Disorder: Protocol for a Phase 1, Open-Label Study

**DOI:** 10.2196/17223

**Published:** 2020-06-04

**Authors:** Arthi Chinna Meyyappan, Roumen Milev

**Affiliations:** 1 Centre for Neuroscience Studies Queen's University Kingston, ON Canada; 2 Providence Care Hospital Kingston, ON Canada; 3 Department of Psychiatry Queen's University Kingston, ON Canada; 4 Department of Psychology Queen's University Kingston, ON Canada

**Keywords:** depression, anxiety, microbial ecosystem therapy, gut-brain axis, microbiome, clinical trial, protocol

## Abstract

**Background:**

The bidirectional signaling between the gut microbiota and the brain, known as the gut-brain axis, is being heavily explored in current neuropsychiatric research. Analyses of the human gut microbiota have shown considerable individual variability in bacterial content, which is hypothesized to influence brain function, and potentially mood and anxiety symptoms, through gut-brain axis communication. Preclinical and clinical research examining these effects suggests that fecal microbiota transplant (FMT) may aid in improving the severity of depression and anxiety symptoms by recolonizing the gastrointestinal (GI) tract with healthy bacteria. The microbial ecosystem therapeutic (ie, microbial ecosystem therapeutic-2 [MET-2]) used in this study is an alternative treatment to FMT, which comprises 40 different strains of gut bacteria from a healthy donor.

**Objective:**

The primary objective of this study is to assess subjective changes in mood and anxiety symptoms before, during, and after administration of MET-2. The secondary objectives of this study are to assess the changes in metabolic functioning and the level of repopulation of healthy gut bacteria, the safety and tolerability of MET-2, and the effects of early stress on biomarkers of depression/anxiety and the response to treatment.

**Methods:**

Adults experiencing depressive or anxiety symptoms will be recruited from the Kingston area. These participants will orally consume an encapsulated MET-2 once daily—containing 40 strains of purified and laboratory-grown bacteria from a single healthy donor—for 8 weeks, followed by a 2-week treatment-free follow-up period. Participants will undergo a series of clinical assessments measuring mood, anxiety, and GI symptoms using validated clinical scales and questionnaires. Molecular data will be collected from blood and fecal samples to assess metabolic changes, neurotransmitter levels, inflammatory markers, and the level of engraftment of the fecal samples that may predict outcomes in depression or anxiety.

**Results:**

Given the association between the gut bacteria and the risk factors of depression, we expect to observe an improvement in the severity of depressive and anxiety symptoms following treatment, and we expect that this improvement is mediated by the recolonization of the GI tract with healthy bacteria. The recruitment for this study has been completed, and the data obtained are currently being analyzed.

**Conclusions:**

This is the first time MET-2 is being tested in psychiatric indications, specifically depression and anxiety. As such, this may be the first study to show the potential effects of microbial therapy in alleviating psychiatric symptoms as well as its safety and tolerability.

**International Registered Report Identifier (IRRID):**

DERR1-10.2196/17223

## Introduction

### Depression and Anxiety

Major depressive disorder (MDD) is the leading cause of disability worldwide and is a major contributor to the overall global burden of diseases [1]. Globally, over 300 million people of all ages are affected by MDD over their lifetime, and the mortality risk of suicide for these individuals is 20-fold greater than that of the general population [2]. MDD is characterized by either a persistent depressed mood or loss of interest or pleasure in surroundings accompanied by a variety of symptoms that cause clinically significant distress or impairments [3]. MDD may have a severe and chronic course, with the majority of affected individuals subjected to multiple recurrent episodes throughout their lifetime as well as academic, occupational, and interpersonal functioning impairments and high levels of medical and psychiatric comorbidities [4].

A common comorbidity in individuals with MDD is generalized anxiety disorder (GAD). GAD is characterized by excessive anxiety and worry, particularly due to life circumstances, such as work, school, and relationships [5]. GAD is one of the most common and the broadest anxiety disorder affecting 3% of the general population in 1 year, with a lifetime prevalence of 5% [6,7]. The psychological symptoms of GAD can also be accompanied by physical symptoms, such as abdominal pain, muscle tension, restlessness, or sleeping problems. The association between generalized anxiety and gastrointestinal (GI) symptoms may be explained by the close connection between the gut and the brain.

### Gut Brain Axis

In the last several years, there has been a growing appreciation for research in the field of the *gut****-****brain axis*, which consists of bidirectional signaling between the GI tract and the brain. Over 100 trillion commensal bacteria are estimated to exist symbiotically in the human gut and are critical in the normal development of the immune system, central nervous system (CNS) circuitry, GI functioning, and autonomic nervous system functioning [8]. Detailed analyses of human gut microbiota have shown considerable individual variability in bacterial content, as this dynamic system is influenced by a variety of factors, such as genetics, diet, metabolism, age, geography, antibiotic treatment, and stress [8]. Studies have also shown that human gut bacteria play a vital role in regulating important aspects of brain development and function, along with other host physiology [9,10].

By being able to shape brain physiology and, therefore, behavior through gut**-**brain axis communication, gut bacteria may be a vital trigger in the development of neuropsychiatric conditions [11]. Given the adaptable nature of the gut microbiome, it may be a good representation of the individual’s history and could explain the differences in risk of illness, disease course, and response to treatment. The interaction of the gut with the environmental risk factors of depression and anxiety, such as diet and early life stress, also suggests that interventions that target the gut microbiome could prevent and treat depression and anxiety symptoms [12]. In past studies, depressed patients have been shown to have a dissimilar microbiota composition compared with healthy individuals due to the decreased diversity and abundance of their gut microbiota [13-15].

### Gut Microbiome and Psychiatric Symptoms

MDD pathophysiology involves three different physiological systems: (1) neurotransmitter dysfunction between the prefrontal cortex regions in the CNS and the hypothalamic-pituitary-adrenal (HPA) axis, (2) chronic inflammation of the immune system, and (3) an imbalance in the microbiota-gut**-**brain axis [16-20]. The prefrontal cortex, hippocampus, and amygdala play a vital role in the regulation of emotion, anxiety, stress responses, self-control, motivation, and cognitive reactions. However, in depressed patients, the function of the prefrontal cortex and hippocampus is impaired, whereas the activity of the amygdala is increased [21]. These arguments are supported by the monoaminergic neurotransmitter deficiency hypothesis, which postulates that monoamine neurotransmitters serotonin (5-HT), norepinephrine (NE), and dopamine (DA) are at insufficient levels and ultimately result in depressive symptoms. The immune system plays an important role in depression; the cytokine and neuroinflammation hypotheses suggest an increase in pro-inflammatory cytokines (interleukin 6 [IL-6] and tumor necrosis factor alpha [TNF-α]), and a decrease in anti-inflammatory cytokines (interleukin 10 [IL-10] and transforming growth factor beta [TGF-β]), contribute to a pro-inflammatory state in depressed patients. These cytokines inhibit the negative feedback of the HPA axis, increase the permeability of the blood-brain barrier, reduce the synthesis of 5-HT, disturb the glutamatergic systems, and influence neuroglial cells. These actions play a vital role in the regulation of neuro-immunity and neuroplasticity and are hypothesized to result in depressive behavior [22-27]. In 2012, Maes et al [28] showed that bacterial translocation from the gut, or *leaky gut*, which may be due to systemic inflammation in depression or may be a primary trigger that is associated with the onset of depression, can activate immune cells to elicit selective immunoglobulin A (IgA) and immunoglobulin M (IgM) response—both antibodies that fight and protect against infections. The results of this study suggest that this phenomenon may be involved in the pathophysiology of depression by causing progressive amplifications of immune pathways [28].

### Microbial Ecosystem Therapeutics

Despite increasing research, a complete understanding of gut microbial changes is still lacking. Interestingly, depressive symptoms have been shown to be transmissible via fecal microbiota transplantation (FMT) in animal models [29]. Zheng et al [30] demonstrated that FMT material from depressed but not neurotypical human patients resulted in depressive-like symptoms upon transfer to germ-free recipient mice. Given that disturbances to the gut microbiome can increase susceptibility to depression, repopulating the gut microbiome may allow for improvement in mood and anxiety symptoms. Microbial ecosystem therapeutic-2 (MET-2) is a new treatment approach for repopulating the gut with healthy bacteria that has been developed as an alternative to FMT. This is a biological compound comprising live microbes that normally reside in the gut of a healthy individual. Given that stool contains a highly complex and dense community of microbes, including bacteria, fungi, and viruses, many of which have not been fully characterized, the product focus of MET-2 is on defined mixtures of isolated strains of intestinal bacteria as the treatment [31]. MET-2 capsules contain 40 strains of purified lyophilized bacteria from a healthy 25-year-old donor, chosen for their favorable safety profile. MET-2 capsules are produced under conditions compatible with good manufacturing practice, at the University of Guelph, sealed under anaerobic conditions, and shipped at room temperature. The vials of MET-2 capsules are transferred at room temperature from the University of Guelph to the Providence Care Hospital pharmacy. The investigator ensures that the product is stored at room temperature ready for use in appropriate conditions and in a secure, locked storage with controlled access until use. Capsules are used to improve MET-2’s appeal to participants to allow for easier administration over consecutive days, in contrast to FMT, which involved of rectal administration. Moreover, capsules allow for oral administration of controlled fecal colonies, which confers an increased level of safety over the use of raw fecal material administered via rectal suspension. This protocol will outline a phase 1, open-label clinical trial that will assess the safety, efficacy, and tolerability of MET-2 on depression and anxiety symptoms.

### Objectives

#### Primary Objective

The primary objective of this study is to assess subjective changes in mood and anxiety symptoms before, during, and after MET-2 treatment in participants with major depression and/or generalized anxiety, using the Montgomery-Asberg depression rating scale (MADRS), GAD 7-item scale (GAD-7), and other mood/anxiety scales. Those with a 50% reduction in the GAD-7 and/or MADRS scores will be considered successful responders to treatment.

#### Secondary Objectives

The secondary objectives are to assess changes in metabolic functioning/levels before, during, and after treatment; to assess safety and tolerability, including adverse events (AEs) with a severity of grade 2 or above; and to assess potential correlation between early life stress (childhood emotional/physical/sexual abuse history, etc) and changes in MDD biomarkers as well as response to treatment.

## Methods

### Study Design

This is an 8-week, open-label, phase 1 clinical trial assessing the safety, tolerability, and efficacy of MET-2 on depression and anxiety symptoms. All participants will consume the investigational product, MET-2, in *a* capsule form, daily for 8 weeks. MET-2 is administered orally at 0.5 g of MET-2 per capsule, containing 3.2 ×10^5^-3.2×10^11^ colony-forming units (CFUs). At each in-hospital visit, participants take home the appropriate number of MET-2 capsules needed for a 2-week period, packaged in bottles. Loading/booster doses are provided in separate vials from maintenance daily doses. Each vial is stored by the participant at room temperature. MET-2 is meant to be taken after a light meal, preferably in the morning and at the same time every day.

Participants will consume an initial loading dose of 5 g MET-2. An initial MET-2 dose in the range of 10^8^ to 10^12^ CFUs was selected with the aim of ensuring delivery of a sufficient quantity of the MET-2 bacterial community that is expected to colonize the gut. The first loading dose, at baseline, is to be consumed under the supervision of the study staff to ensure immediate safety, and further compliance is assessed using the returned investigational product and by reviewing participants’ personal logs at each in-hospital visit. For the remainder of the 2-week period, the participants will consume 1.5 g of MET-2 per day. The loading dose is repeated for the first 2 days at the week 2 time point, and participants return to the maintenance dose for the remainder of the treatment period. At week 4, participants are assessed for treatment response. Responders remain on the maintenance dose until the end of treatment. Nonresponders are given an additional loading dose (booster dose; Table 1).

**Table 1:** Dosing schedule.

Dose	Baseline to week 2	Weeks 2*-*4	Weeks 4*-*6	Weeks 6*-*8
Loading/booster dose (5 g of MET-2^a^: 10 capsules per day for 2 days)	Days 1 and 2 only	Days 1 and 2 only	Days 1 and 2 (only NR^b^)	N/A^c^
Maintenance dose (1.5 g of MET-2: 3 capsules per day)	Days 3-14	Days 3-14	R^d^: Days 1-14; NR: Days 3-14	Days 1-14

^a^MET-2: microbial ecosystem therapeutic-2.

^b^NR: nonresponders.

^c^N/A: not applicable.

^d^R: responders.

### Definitions

#### Depression/Presence of Depressive Symptoms

The diagnosis of MDD is based on the mini-international neuropsychiatric interview (MINI). A score of ≥15 on the MADRS is also used to diagnose MDD.

#### Anxiety/Presence of Anxiety Symptoms

The diagnosis of GAD is based on MINI. A score of ≥8 on the GAD-7 is also used to diagnose GAD.

#### Treatment Success

Treatment success is defined as a reduction in the MADRS/GAD-7 scores by 50% from the baseline. Those who do not meet the 50% threshold are considered nonresponders. Nonresponders at the week 4 visit will be given a booster dose.

#### Treatment Failure

Treatment failure is defined as same or increased MADRS/GAD-7 scores from pre- to post-treatment.

### Study Setting

This study is being carried out at the Providence Care Hospital, a tertiary care mental health and continuing care hospital in Kingston, Ontario There will be a total of 7 visits, of which 6 will be in-person and 1 will be over the phone.

### Participants

A total of 10 to 15 eligible participants aged between 18 and 65 years will be recruited from the Kingston area. At the first visit, consent will be obtained from potential trial participants by a study team member and overseen by the principal investigator. Following informed consent, the participants will be screened to ensure that they fit the inclusion criteria (Textbox 1). In this study, we define depression as a confirmed diagnosis of MDD based on the MINI and a score of ≥15 on MADRS; anxiety is defined as a confirmed diagnosis of GAD based on the MINI and a score of ≥8 on the GAD-7. These participants are not to be in a current episode of MDD or GAD and should not have been taking any antidepressant medication. Textboxes 1 and 2 provide the full study criteria.

**Inclusion criteria.**

- Able to provide informed consent
- Not pregnant
- Willing to participate in follow-up as part of the study
- Diagnosis of major depressive disorder and/or generalized anxiety disorder (GAD) by the mini-international neuropsychiatric interview
- Current depressive episode with a Montgomery-Asberg depression rating scale score of ≥15 or current GAD episode with a GAD 7-item scale score of ≥8
- Able to understand and comply with the requirements of the study
- Able to provide stool and blood samples

**Exclusion criteria.**

- History of chronic diarrhea
- Need for regular use of agents that affect gastrointestinal motility (narcotics such as codeine or morphine and agents such as loperamide or metoclopramide)
- Colostomy
- Elective surgery that will require preoperative antibiotics planned within 6 months of enrollment
- Pregnant, breastfeeding, or planning to get pregnant in the next 6 months
- Any condition for which, in the opinion of the investigator, the participant should be excluded from the study
- Current use of any antidepressant/antianxiety drug (eligible to participate after a 4-week washout period)
- Use of any antibiotic drug in the past 4 weeks (may be eligible to participate after a 4-week washout period)
- History of alcohol or substance dependence in the past 6 months
- Daily use of probiotic products in the past 2 weeks (may be eligible to participate after a 2-week washout period)
- Use of any type of laxative in the last 2 weeks
- Consumption of products fortified in probiotics

### Recruitment

Participants were recruited from the Kingston community using web-based and paper advertisements. Web-based advertisements were posted on the internet to social media platforms such as Facebook and Twitter. Posters were posted around Kingston—on university and college campuses, community bulletin boards, clinics, and counsellor centers. Recruitment was completed in December 2019.

### Treatment Compliance

Treatment compliance was assessed by recording unused clinical trial material and reviewing participants’ personal logs.

### Discontinuation Criteria

A participant may be discontinued from treatment or withdrawn from the study at any time if the participant, the principal investigator, or the investigational product sponsor does not feel that it is not in the participant’s best interest to continue. The following is a list of possible reasons for treatment discontinuation:

- Participant’s withdrawal of consent
- An AE that in the opinion of the investigator would be in the best interest of the participant to discontinue the study treatment
- Protocol violation requiring discontinuation of the study treatment
- Lost to follow-up
- Request by NuBiyota LLC for early termination of the study
- Newly pregnant participants.

If a participant is withdrawn from treatment due to an AE, the participant will be followed up and treated by the investigator until the abnormal parameter or symptom has resolved or stabilized. All participants are free to withdraw from participation at any time, for any reason, specified or unspecified, and without prejudice. Reasonable attempts will be made by the investigator to provide a reason for the withdrawal of participants. No further data will be collected once a participant has been withdrawn.

### Outcome Measures

#### Clinical Measures

Mood, anxiety, and GI symptoms and sleep quality will be assessed at all treatment visits. At week 10, only the MADRS and GAD-7 will be administered. The primary outcome measure will be mood and anxiety symptoms measured biweekly by changes in the MADRS and GAD-7 scores, respectively. The clinical measures include self-rated and clinician-rated measures. The self-rated questionnaires that will be used are the GAD-7 to assess anxiety symptoms and severity, the Snaith-Hamilton pleasure scale to assess anhedonia, quick inventory of depressive symptomatology to assess depressive symptoms, the gastrointestinal symptom rating Scale (GSRS) to assess GI symptoms, the Toronto side effects scale (TSES) to assess tolerability of the therapeutic product, and the Pittsburgh sleep quality index to assess subjective sleep quality. The clinician-rated questionnaires and interviews are the MADRS to assess depressive symptoms and severity, the clinical global impressions scale to assess illness severity and improvement, and the childhood experience of care and abuse questionnaire to assess early life stress and environmental risk factors associated with upbringing. Table 2 gives more details on the scales used in assessing outcome measures.

**Table 2:** Study visit schedule.

Procedure		Screening	Week 0	Week 2	Week 4	Week 6	Week 8	Week 10
**Clinical**								
	ICF^a^ review and signing	X^b^	—^c^	—	—	—	—	—
	Study criteria review	X	—	—	—	—	—	—
	Mini-international neuropsychiatric interview	X	—	—	—	—	—	—
	Demographics	X	—	—	—	—	—	—
	Medical and antidepressant history	X	—	—	—	—	—	—
	Physical examination	—	X	—	—	—	—	—
	Pregnancy test (females)	—	X	—	—	—	—	—
	Montgomery-Asberg depression rating scale	X	X	X	X	X	X	X
	Generalized anxiety disorder 7-item scale	X	X	X	X	X	X	X
	Clinical global impressions scale	—	X	X	X	X	X	—
	Snaith-Hamilton pleasure scale	—	X	X	X	X	X	—
	Quick inventory of depressive symptomatology	—	X	X	X	X	X	—
	Pittsburgh sleep quality index	—	X	X	X	X	X	—
	Toronto side effects scale	—	X	X	X	X	X	—
	Gastrointestinal symptom rating scale	—	X	X	X	X	X	—
	Childhood experience of care and abuse	—	—	—	—	X	—	—
	Adverse events	—	X	X	X	X	X	X
**Molecular**								
	Blood samples	X	—	—	X	—	X	—
	Fecal samples	—	X	X	X	—	X	X

^a^ICF: Informed Consent Form.

^b^X: will be completed.

^c^Will not be completed.

#### Safety and Tolerability

Participants will use a personal mood and symptom log to track any symptoms that they have been newly experiencing since the beginning of treatment, assess the tolerability of treatment, and keep track of their mood and sleep. AEs will be assessed and recorded at all in-hospital visits and on phone calls; they will be categorized by frequency, severity, and causality. The frequencies and severity of AEs will be collected on a questionnaire from the first day of the assigned study treatment through 14 days following the last assigned study treatment. Investigational product safety will be assessed via recorded symptoms and AEs. Investigational product tolerability will also be assessed using the TSES and GSRS during all visits except screening.

### Molecular Analysis

Blood samples will be taken at screening, *w*eek 4, and *w*eek 8 and sent to Core Laboratories at Kingston Health Sciences Centre, Kingston General Hospital to analyze changes in cortisol levels, liver function, lipids, inflammatory markers (cytokines IL-10 and IL-6, TNF-α, TGF-β, and C-reactive protein), neurotransmitters (5-HT, *DA*, and *NE*), and immunoglobulins (IgA, IgG, and IgM). Baseline levels of creatinine, electrolytes, thyroid-stimulating hormone, complete blood count, and glucose will be taken only at screening to ensure that it is safe for participants to consume the investigational product at baseline. Fecal samples will be collected at all in-hospital visits. The participants will be provided with a stool kit and a Styrofoam case, with instructions at each visit. Upon collecting the sample from the participants, it will be stored at –62°C. Throughout the study, the samples will be sent to the University of Guelph for 16s RNA gene sequencing to determine the gut microbiome composition before, during, and after treatment.

### Data Management

Data will be collected on case report forms and source documents pertaining to the questionnaires mentioned earlier in this paper and stored at the Providence Care Hospital. It will then be entered into a secure web-based database at the Providence Care Hospital and will only be accessible by the study team. To ensure confidentiality during the study, subject data will be analyzed under a randomized assigned study number. Participants’ study files will be kept in a designated locked room, only accessible by the study co-coordinator and study investigators for 25 years, as per the International Council for Harmonisation of Technical Requirements for Pharmaceuticals for Human Use (ICH) guidelines.

Blood and fecal samples will be stored at the Providence Care Hospital until the time of transfer, which will occur intermittently throughout the study. A material transfer agreement will be in place as per the Health Sciences and Affiliated Teaching Hospitals research ethics board (HSREB) protocol for all biological specimen transfers.

### Statistical Analyses

Treatment success and failure will be measured individually. Those who have an improvement of at least 50% in MADRS or GAD-7 scores will be considered responders. The SPSS will be used to analyze all data from clinical measures obtained throughout the study. Repeated measures analysis of variance (ANOVA) will be used to analyze changes in clinical measures from baseline to week 10. Paired *t* tests will be used to compare clinical measure means at each time point with the baseline. If a participant returns after the first course of treatment and is later withdrawn, their final scores for primary outcomes will be projected to week 10. In the event of missing data, the data from the last time point will be projected forward. Similarly, stool samples will be analyzed to provide diversity scores, which will then be compared using paired *t* tests and repeated measures ANOVA. Changes in diversity scores will be compared with clinical scores to assess for correlations.

### Data Monitoring

Monitoring visits will be conducted by representatives of NuBiyota LLC according to the ICH guidelines for good clinical practice (GCP; E6). All information obtained during the course of this study is strictly confidential, and each subject’s anonymity will be protected at all times. The investigator grants permission to NuBiyota LLC (or designee) and appropriate regulatory authorities to conduct on-site monitoring and/or auditing of all appropriate study documentation. Subjects will be identified by a study number and will not be identified in any publication or reports. The information collected for the study will be kept in a locked and secure area by the study doctor for 25 years. Only the study team, the representatives of the Research Ethics Board, and the representatives of Health Canada will have access to the data.

In addition, the study data may be transferred to the people or groups listed above to be processed for the purposes of the study, for product registration purposes and scientific purposes in general, ensuring compliance with laws and regulations. Any study data transferred outside of this study site will not include the name, address, or health insurance number of the subject. Instead, the study data will be coded to ensure that each subject’s identity is kept confidential.

### Adverse Events

The frequencies and severity of AEs will be collected on a questionnaire from the first day of the assigned study treatment through 14 days following the last assigned study treatment. AEs will be assessed and recorded at all in-hospital visits and phone calls; they will be categorized by frequency, severity, and causality.

#### Severity

To rate the severity of AEs, the following 4-point rating scale will be used:

1. Life-threatening (4 points): The subject is at risk of death due to adverse experiences as it occurred. This does not refer to an experience that hypothetically might have caused death if it was more severe.
2. Severe (3 points): Signs or symptoms result in a complete inability to pursue regular activities.
3. Moderate (2 points): Signs or symptoms disrupt regular activities.
4. Mild (1 point): Signs or symptoms present but do not disrupt regular activities.

#### Causality

To gauge the causal relationship of the AEs to the treatment, the following 4-point scale will be used:

1. Probable (4 points): A clear-cut temporal association with improvement on cessation of investigational medicinal products or reduction in dose. Reappears upon rechallenge.
2. Possible (3 points): Follows a reasonable temporal sequence from administration. May have been produced by the subject’s clinical state, environmental factors, or other therapies administered.
3. Unlikely (2 points): Does not follow a reasonable temporal sequence from administration. May have been produced by environmental factors or other therapies administered.
4. Unrelated (1 point): Due only to extraneous causes and does not meet the criteria listed under unlikely, possible, or probable.

The investigator will probe, via discussion with the participant, for the occurrence of AEs during each patient visit and record the information in the site’s source documents. AEs will be recorded in the participant case report form. AEs will be described by duration (start and stop dates and times), severity, outcome, treatment, and relation to the study drug or, if unrelated, the cause.

AEs will be collected in the following ways: (1)any symptoms or events self-reported spontaneously by the patient,

(2) the patient will be asked open-ended questions to answer about their health (eg, “How are you feeling since we saw you last in clinic?”), and (3) any clinically relevant abnormalities noted by the investigator during follow-up interviews.

In the event of a reported AE, if the principal investigator decides that it would be in the best interest of the participant to discontinue treatment, the participant will be removed from the study. If a participant is withdrawn from treatment due to an AE, the participant will be followed and treated by the investigator until the abnormal parameter or symptom has resolved or stabilized.

Once withdrawn, if participants choose to withdraw the biological samples they provided before the AE, they are able to do so, and we will ensure that the samples are destroyed. If tests have already been performed on the sample(s), it will not be possible to withdraw those results. However, no further testing will be performed. The samples that have already been tested will continue to the analysis portion of the study. 

### Serious Adverse Events

Serious adverse events (SAEs) will also be reported to the HSREB and given immediate care. SAEs are any untoward medical occurrence that at any does results in death, is life-threatening, requires inpatient hospitalization or prolongation of existing hospitalization, results in persistent or significant disability/incapacity, or is a congenital anomaly/birth defect. 

### Requirement to Report

AEs that are considered serious, unexpected, and possibly or probably related to the study drug are subject to expedited reporting to the REB and Health Canada in accordance with C.05.014 of the Canadian Food and Drug Regulations. It is the responsibility of the investigator to report serious, unexpected, and possibly or probably related AEs to the REB. It is the responsibility of NuBiyota LLC to report serious, unexpected, and possibly or probably related AEs to Health Canada. 

### Reporting Procedures

After learning that a participant has experienced an SAE, the investigator or designee is responsible for reporting the SAE to the medical monitor, regardless of relationship or expectedness, within 24 hours of becoming aware of the event.

This report may be accomplished by completing an SAE form, which will include the following:

- Subject’s study number
- Subject’s gender
- Date of first dose of investigational drug(s)
- Date of last dose of investigational drug(s), if applicable
- AE term
- Time and date of occurrence of the event
- A brief description of the event, outcome to date, and any actions taken
- The seriousness criteria (on) that were met
- Concomitant medication at the onset of the event
- Relevant medical history information
- Relevant laboratory test findings
- Investigator’s opinion of the relationship to investigational drug(s) (“Is there a reasonable possibility that the investigational drug caused the SAE? Yes or No?”).

### Approvals and Registration

This trial will be conducted in compliance with the protocol, GCP, and the applicable local regulatory requirements and laws. Ethics approval was obtained from Queen’s University HSREB (protocol number 6025187) on March 28, 2019. A no-objection letter from Health Canada was obtained for the use of MET-2, in accordance with part C, division 5 of the Food and Drug Regulations. The trial was registered on ClinicalTrials.gov on August 9, 2019 (NCT04052451), and is being funded by NuBiyota LLC and MITACS.

If any changes are made to the study protocol by the investigator, such as changes to eligibility criteria, informed consent, outcomes, and/or analyses, they will be communicated to the sponsor, the HSREB, Health Canada, and trial registry. If required, all participants will be informed and consented once again.

### Access to Data

The investigator and team will have access to the final trial dataset. This has been outlined in a clinical trial agreement and has been agreed upon by both the investigator and the sponsor. Upon completion of trial and data analysis, the study results will be published for participants, health care professionals, the public, and all other relevant groups to access. The publications will be written and authored by the principal investigator and team.

## Results

The study received approval from the Queen’s University Health Sciences and Affiliated Teaching Hospital Research Ethics Board on March 28, 2019. Participant recruitment began in May 2019. As of March 2020, we have enrolled 21 participants, of which 12 participants completed the study with at least one visit postbaseline. We have completed recruitment for this study and are currently in the process of analyzing data. We expect to see improvement in depressive and anxiety symptom scores from baseline to week 10, to be mediated by a decrease in pro-inflammatory markers and an increase in anti-inflammatory markers. Furthermore, we hope to see lasting changes in the microbiome composition reflecting a similar composition to that of the donor by the end of the study.

## Discussion

Currently, there are many different psychological and pharmacological treatments that target symptoms of depression and anxiety as well as other emerging novel treatment methods. However, progress in research on MDD and GAD has been challenging due to high individual variability in symptoms and course, high levels of comorbidity with other psychopathology, and genetic *and* environmental influences. Given these challenges, identifying novel and personalized methods is crucial for advancements in research on depression and anxiety. The therapeutic link between MDD/GAD and gut microbiota is advantageous because of the greater accessibility and modifiability of the microbiome compared with the human genome [32]. Given the adaptable nature of the microbiome, it may be a good representation of the individual’s history and could explain the differences in risk of illness, disease course, and response to treatment. If MET-2 alleviates symptoms of neuropsychiatric disorders, they could be offered to patients as personalized, alternative, and/or adjunctive treatments to combat specific symptoms that tie together specific gut bacteria strains or the gut as a whole to the brain.

However, possible limitations of this trial include difficulty recruiting individuals with depression or anxiety that are not currently on any antidepressant medication or due to stigma associated with fecal and/or natural products. Compliance with the treatment schedule can also be more difficult for individuals with mental illness [33]. These issues will be minimized by widespread advertising and a detailed explanation of study requirements before signing the informed consent form. Participants will also be asked to keep a mood and symptom chart where they will also track their investigational product use. A researcher from the study team will discuss the details of the chart at each in-hospital visit to assess potential adverse symptoms and maintain a record of investigational product compliance. Given that this is an open-label trial, other limitations include small sample size, lack of a placebo arm, and a short follow-up period.

In summary, to our knowledge, this will be the first study to show evidence for the role of microbial therapy in treating depression and anxiety. Furthermore, the results of the trial will contribute to a growing body of research on assessing gut repopulation as a potential treatment method for MDD and GAD and could further explain the relationship between the gut and the brain and the underlying mechanisms.

## Appendix

Multimedia Appendix 1 Peer-reviewer report from MITACS.

## Abbreviations

5-HT: serotonin
AEs: adverse events
ANOVA: analysis of variance
CFU: colony-forming unit
CNS: central nervous system
DA: dopamine
FMT: fecal microbiota transplantation
GAD: generalized anxiety disorder
GCP: good clinical practice
GI: gastrointestinal
GSRS: gastrointestinal symptom rating scale
HPA: hypothalamic-pituitary-adrenal
HSREB: Health Sciences and Affiliated Teaching Hospitals research ethics board
ICH: International Council for Harmonisation of Technical Requirements for Pharmaceuticals for Human Use
IgA: immunoglobulin A
IgM: immunoglobulin M
IL-10: interleukin 10
IL-6: interleukin 6
MADRS: Montgomery-Asberg Depression Rating Scale
MDD: major depressive disorder
MET-2: microbial ecosystem therapeutic-2
MINI: mini-international neuropsychiatric interview
NE: norepinephrine
SAEs: serious adverse events
TGF-β: transforming growth factor beta
TNF-α: tumor necrosis factor alpha
TSES: Toronto side effects scale
