# Supplementary material for: The Safety, Efficacy, and Tolerability of Microbial Ecosystem Therapeutic-2 in People With Major Depression and/or Generalized Anxiety Disorder: Protocol for a Phase 1, Open-Label Study
Source: JMIR Res Protoc. 2020 Jun 4;9(6):e17223. doi: 10.2196/17223 (PMC7303825; doi:10.2196/17223)
Supplement: Multimedia Appendix 1 [file resprot_v9i6e17223_app1.pdf]

July 15, 2019

Application Ref. IT15330

RE: Mitacs Accelerate Proposal

**Project Title:**	**The Safety, Efficacy, and Tolerability of Microbial Ecosystem Therapeutic-2 in People with Major Depression and/or Generalized Anxiety Disorder**
**Internship Supervisor(s):**	**Roumen Milev**
**Department:**	**Department of Psychiatry**
**Institution:**	**Queen's University**
**Partner Organization:**	**NuBiyota**

Dear applicants,

Your recent proposal for a Mitacs Accelerate internship project has been reviewed. Based on this review, we request that you submit a revised proposal addressing the following comments **(see Appendix A).**

Please address the comments in your revised submission so that we may continue considering your proposal for funding. Please include a cover letter indicating your response to any specific points and highlight any changes to your proposal so they are easily identifiable. We look forward to receiving your revised proposal.

Please note that you have **45 days** to respond to the request for revisions. If your revised proposal is received by Mitacs more than 45 days after the date of this letter, it will be treated as a new application.

If you have any questions about this letter, you may contact the Chair of the Mitacs Research Council at mrc-chair@mitacs.ca.

Yours truly,

R. Ben Mrad

Ridha Ben Mrad
Chief Research Officer and Associate Academic Director

Appendix A: Referee Comments

Your application has been reviewed by three external experts. Overall, the reviewers have found the project interesting and challenging. However, the reviewers have raised concerns about the objectives, the methodology and the timeline, that we would like you to address in a revised version of the proposal. In particular, we would like you to clarify some aspects of the protocols, to justify the design of the study, and to update or discuss your timeline to make sure that all the objectives will be achieved in the allotted time. For the revisions, please refer to the comments from Reviewer 1 - questions #3-5, Reviewer 2 - questions #4-6 and Reviewer 3 - questions #4 & 6.
The full reviewer reports are provided for your consideration.

Reviewer 1

1) Mitacs Accelerate supports research-based internships. Does the proposed project qualify as research in its discipline?
The proposed project qualifies as research in the discipline specified in the proposal (Life Sciences) as well as the disciplines of the MSc applicant (Neuroscience) and her supervisor (Neuroscience, Psychiatry). Briefly, the project will test the effects of a 8-week treatment with a new Microbial Ecosystem Therapeutic (MET-2; composed of 40 strains of freeze-dried bacteria isolated and purified from a 25-year old donor), developed by the industrial partner NuBiyota, on severity of depressive and anxiety symptoms in drug-free participants with major depressive disorder or generalized anxiety disorder. Gastrointestinal symptoms, quality of sleep, history of early-life stress and tolerability to MET-2 as well as blood levels of cortisol, lipids, glucose, inflammatory cytokines, neurotransmitters and immunoglobulin and fecal bacterial composition will also be investigated throughout the treatment. It is expected that MET-2 will improve mood and anxiety in the study participants and that these effects will be mediated by a microbiota shift where healthy bacteria from MET-2 will colonize the host's gut environment. This is a very timely and promising project and findings from this research may make substantial contributions in the growing field of microbiota-gut-brain axis and mental health and provide new opportunities of treatment in people with depression and/or anxiety.

2) Is the project appropriate for the academic degree level of the intern(s)?
The proposed project is appropriate for an intern at the MSc level provided the intern will be receiving proper guidance and support throughout the study, especially to conduct the several planned analyses (which seems to be the case). Recruitment of individuals with mood and anxiety disorders, especially their retention for 10 weeks (including 8 weeks of treatment) in the study, can be challenging. The use of different types of assays to conduct the proposed blood and fecal analyses can also be very demanding. The fact that the industrial partner will provide the equipment and expertise to analyse biological samples is an important advantage and will help the intern to face some of these challenges.

3) Are the objectives clear?
The main objective of the proposed research is to assess changes in mood and anxiety symptoms in individuals with major depression or generalized anxiety throughout exposure to the new product MET-2. Secondary objectives will examine potential adverse effects of MET-2 as well as changes in fecal microbiota composition (mainly to confirm microbial engraftment/efficacy of bacterial colonization) and in blood levels of cortisol, metabolic/inflammatory factors and neurotransmitters throughout MET-2 treatment. A last objective will also examine the relationships between early-life stress and response to treatment as well as MDD biomarkers.

The main and secondary objectives are fairly clear - basically the efficacy of a new microbiota-based intervention in improving mood and anxiety symptoms will be tested among drug-free depressed and anxious individuals and a series of biological factors in blood will be examined as potential contributors to these symptom improvements (in addition to microbial engraftement).
In relation to the 3rd secondary objective, however, it is not entirely clear why relationships between early-life trauma and biomarkers will be investigated in MDD participants only (page 6, section 2.4)?
4) Is the methodology appropriate to achieve the objectives?
The methodology described to conduct the proposed research project appears appropriate. The MINI is one of the gold standards to identify mood and anxiety disorders (here major depression and generalized anxiety), the study questionnaires to assess depression/anxiety symptoms are adequate (e.g., MADRS, GAD-7) as well as the scales for sleep quality and early-life stress. The use of the 16S assay to assess fecal microbiota composition (and thus microbial engraftement) is also appropriate. Unfortunately, however, no details are provided on the collection procedures and the type of assays that will be used to quantify levels of metabolic/inflammatory factors, and most importantly neurotransmitters, in the blood. Dopamine, serotonin, and noradrenaline can be quite challenging to quantify in blood (better results are typically observed in urine) and most often their metabolites are quantified instead to achieve more reliable results. If this is feasible, I would suggest to quantify monoamine levels in urine as well.
I understand from the proposal that analyses of biological specimen will be completed within the facilities of the industrial partner (by the intern under their guidance of the partner) and thus I am assuming they have the required equipment and expertise with the assays that will be used. What that said, although the methodology in general appears adequate, I feel it would have been important to specify how biological factors will be assayed to be in a better position to assess if all aspects of the methodology are appropriate.

5) Is the timeline realistic?
The timeline seems a little bit tight to complete all the steps and assays described in the proposed project. It is not specified in the proposal how many participants with major depression and generalized anxiety the applicants are expecting to recruit during the proposed Months 1-8 of the internship (termed recruitment and data collection stages). To achieve enough statistical power with all the proposed analyses (especially correlationnal analyses described in the secondary objectives), I am not sure 8 months is enough to recruit enough participants. With that said, as data will be assayed and analyzed "as samples are collected", this may be a way to overcome this limitation but I think this may be challenging to complete the whole study in the proposed 1-year time frame.

6) Other comments and suggestions for the applicants. For example, is there prior work that should be considered by the researchers?
None.

Reviewer 2

1) Mitacs Accelerate supports research-based internships. Does the proposed project qualify as research in its discipline?
Yes.

2) Is the project appropriate for the academic degree level of the intern(s)?
Yes, this project would be good for training an interns provided the intern is doing the clinical assessments and then also participating in the sample analysis.

3) Are the objectives clear?
Yes, the overall objective is to give a new commercial product that is "purified" bacterial from a 25 year old healthy subject. This product will be given for 8 weeks to determine the clinical effect on depressive and anxiety symptoms. Additionally blood and fecal samples will be obtained to examine the mechanism behind any effect seen.

4) Is the methodology appropriate to achieve the objectives?
I'm not sure. Within the proposal, it states that the subjects will be given first a booster dose and then a second booster dose at 2 weeks. Between these times a standard dose will be given. It was not clear why the second booster dose was needed. Additionally non responders will be given a third booster dose at 4 weeks, but the definition of non-response at this time point was not included. The overall responded definition was a 50% drop in MADRS or GAD-7. It is unclear what happens if subjects meet one of these criteria but not the other. Four weeks may be a little soon to expect a true response, so there should be a better definition of response at 4 weeks if subjects are to be given a booster dose.
The proposal also states that blood and fecal samples will be taken at 3 time points and 5 time points, but it is not clear when these time points are. I'm assuming one will be at the beginning of the study and one at 8 weeks, but when will the others be taken and will they be taken for everyone? Is the 5 time point criteria for the non-responders? There is also mention of a 10 week time period for the study, so overall the study design is very uncertain.
Finally there is no mention of how many subjects will be recruited or how many subjects need to be recruited for this study. Having done microbiome work myself, I would assume that at least 30 subjects would need to be recruited. However that is a guess as I am uncertain what exactly is in the commercial product. Also the inclusion and exclusion criteria are missing from the proposal. Again, given my experience doing this type of work requires a lot of exclusion criteria which makes subject recruitment very difficult.

5) Is the timeline realistic?
I cannot answer this without knowing the inclusion and exclusion criteria or the number of subjects to be recruited. However 8 months seems a bit ambitious. Also four months for the data analysis will be challenging as you will not get results back for at least a month or so, as it is best if the samples are all run at the same time.

6) Other comments and suggestions for the applicants. For example, is there prior work that should be considered by the researchers?
Having additional information about what is in the commercial product and if this matches up with the current microbial data done in depression/anxiety would be helpful to understanding this proposal.
May also consider adding a dietary assessment just to make sure that subjects don't also change their dietary habits during the study. A simple 24 hour food recount done weekly would help.

Reviewer 3

1) Mitacs Accelerate supports research-based internships. Does the proposed project qualify as research in its discipline?
Yes, this is a research project.

2) Is the project appropriate for the academic degree level of the intern(s)?
Yes, the project is a suitable level of investigative science for in intern

3) Are the objectives clear?
Considerable background is given justifying the project in terms of the importance of the gut-brain axis.
The outline of the project (impact of a microbial therapeutic in depressed patients) is very clear.
The primary objective relies on subjective answering of the patients to various questionnaires following treatment.
Secondary objectives include investigating changes in metabolic function by analysis of collected samples; assessing the tolerability and safety of the product and a third objective looking at links with early life stress .

4) Is the methodology appropriate to achieve the objectives?
The primary objective is achievable and a range of validated questionnaire will be used to investigate the patients mood and anxiety symptoms. A concern however is that no allowance is made for a placebo effect. It may well be that simple enrolling on the study and interacting with the researchers on a regular basis will help patients to feel better and thus may improve their scores in the questionnaires. Ideally a placebo capsule, containing only a carrier material and no bacteria would be used in a blinded control in a cross-over study design.
The first two secondary objectives are achievable and seem to have been well considered (although it is not clear how tolerability will be assessed outside GI scores which again could be affected by study enrollment). The researcher mention in the further details (2.5b) that they will assess the level of engraftment of the MET-2 treatment. It is not clear why this is not included in the secondary objectives? If the aim is to illustrate that the improvement in MDD symptoms is due to the MET-2 treatment surely it is important to illustrate a lasting change in the microbiota - either in composition or function? Although engraftment is clearly part of the research it is not sufficiently highlighted.
It is not at all clear how the researchers plan to complete the third secondary objective. No details are given about collecting data on early life stress - will this material be ethically accessed by the researchers? how will 'changes' in MDD markers even be measured - presumably the same questionnaires are not available form individuals prior to the early life stress which would be required. The only achievable art of this objective in my opinion would be the change in MDD markers following treatment - which matches the primary objective.

5) Is the timeline realistic?
Yes. The 8 week duration of the study is sensible, not much detail is given on the remainder of the timeline but it should be established whether or not ethical approval will be in place prior to the start of the study as this could delay the start significantly.

6) Other comments and suggestions for the applicants. For example, is there prior work that should be considered by the researchers?
Get ethical approval in place before commencement of the 1 year period. At the very least an indication should be given of how long this is expected to take based on previous experience.
Think about changing the study design to include a placebo arm - this would lengthen the study but make the findings much more robust. I am concerned about the effect of simply being on the study will have on those recruited, even without treatment.
Have the researchers determined how many patients need to be enrolled in the study to obtain meaningful data? No number is given but there is sufficient pre-existing/published data that they must have an idea of how many individuals they need to enroll in the study to see an effect. This should be added to the proposal outline - there is no point at all in carrying out the study if they have not done a power calculation to support the sample size.

It may be that for the MET-2 engraftment to be more effective that the patient should change their diet to encourage growth and persistence of the 'new' bacterial members of their gut ecosystem. Has this been considered? Since MET-2 has long been studied by the research team they are presumably well aware of substrate requirements by the specific bacterial components. Introducing a dietary change may improve the efficacy - although I appreciate it effectively also requires a control arm of diet change only.
